# Regulation of glucose uptake and inflammation markers by FOXO1 and FOXO3 in skeletal muscle

**DOI:** 10.1016/j.molmet.2018.09.011

**Published:** 2018-11-16

**Authors:** Leonidas S. Lundell, Julie Massart, Ali Altıntaş, Anna Krook, Juleen R. Zierath

**Affiliations:** 1 Department of Physiology and Pharmacology, Section for Integrative Physiology, Karolinska Institutet, Stockholm, Sweden; 2 Department of Molecular Medicine and Surgery, Section for Integrative Physiology, Karolinska Institutet, Stockholm, Sweden; 3 Novo Nordisk Foundation Center for Basic Metabolic Research, Faculty of Health and Medical Sciences, University of Copenhagen, Copenhagen, Denmark

**Keywords:** Skeletal muscle, Glucose uptake, FOXO, Transcriptional regulation, Inflammation

## Abstract

**Objective:**

Forkhead box class O (FOXO) transcription factors regulate whole body energy metabolism, skeletal muscle mass, and substrate switching. FOXO1 and FOXO3 are highly abundant transcription factors, but their precise role in skeletal muscle metabolism has not been fully elucidated.

**Methods:**

To elucidate the role of FOXO in skeletal muscle, dominant negative (dn) constructs for FOXO1 (FOXO1dn) or FOXO3 (FOXO3dn) were transfected by electroporation into mouse *tibialis anterior* muscle and glucose uptake, signal transduction, and gene expression profiles were assessed after an oral glucose tolerance test. Results were compared against contralateral control transfected muscle.

**Results:**

FOXO1dn and FOXO3dn attenuated glucose uptake (35%, *p* < 0.01 and 20%, *p* < 0.05), GLUT4 protein (40%, *p* < 0.05 and 10%, *p* < 0.05), and subunits of the oxidative phosphorylation cascade. Intramuscular glycogen content was decreased (20%, *p* < 0.05) by FOXO3dn, but not FOXO1dn. Transcriptomic analysis revealed major pathways affected by FOXO1dn or FOXO3dn revolve around metabolism and inflammation. FOXO1dn increased Akt protein (140%, *p* < 0.001), *p*-Akt^Ser473^ (720%, *p* < 0.05) and p-Akt^Thr308^ (570%, *p* < 0.01), whereas FOXO3dn was without effect. FOXO1dn and FOXO3dn increased mTOR protein content (170% and 190%, *p* < 0.05), and p-p70S6K^Thr389^ (420%, *p* < 0.01 and 300%, *p* < 0.01), while p-mTOR^Ser2448^ (500%, *p* < 0.01), was only increased by FOXO1dn. Chemokines and immune cell markers were robustly upregulated in skeletal muscle following the FOXOdn transfections, but not after control transfection.

**Conclusions:**

FOXO1 and FOXO3 regulate glucose metabolism and markers of inflammation in skeletal muscle, implicating transcriptional control governing “*immunometabolic*” dynamics.

## Highlights

• FOXO1 and FOXO3 transcriptional activity regulates glucose uptake and inflammation.
• Inhibiting FOXO1 transcriptional activity affects more genes compared to FOXO3.
• Inhibiting FOXO1 and FOXO3 leads to similar pathway enrichment.

## Graphical abstract

FOXO1 or FOXO3 dominant negative (FOXOdn) transfection of tibialis anterior skeletal muscle decreased glucose uptake and protein abundance of GLUT4 and oxidative phosphorylation enzymes. Inflammatory signaling was upregulated by FOXOdn transfection, indicating that FOXO transcriptional activity regulates energy metabolism and inflammatory signaling in skeletal muscle.

**Figure:**
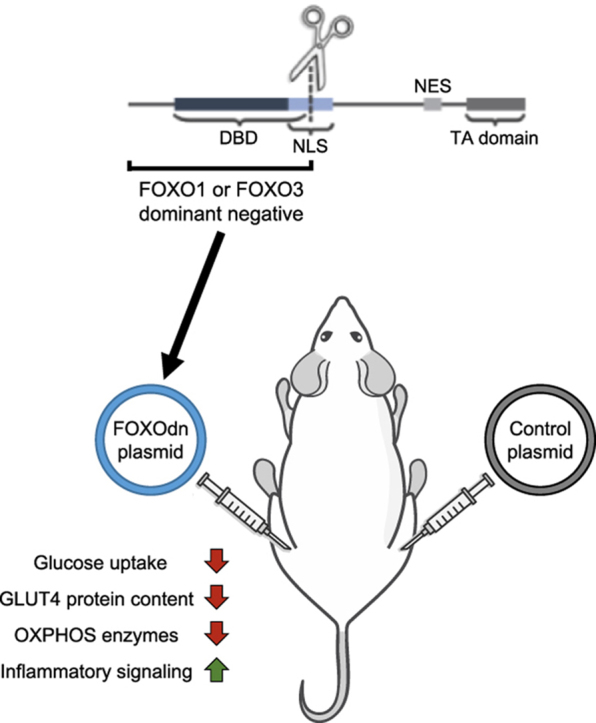

## 1 Introduction

Skeletal muscle is the main insulin-sensitive tissue for postprandial glucose disposal and for the oxidation of glucose- and lipid-based fuels at rest and during exercise [1], [2]. Skeletal muscle is also a site of insulin resistance in type 2 diabetes [3], [4]. Accordingly, skeletal muscle exerts profound effects on whole-body substrate dynamics, as well as playing a vital role in energy expenditure [5]. Type 2 diabetes shares many features of “accelerated aging” including insulin resistance, defective oxidative metabolism/mitochondrial function, and loss of muscle mass [6]. Thus, targeting the transcriptional machinery to maintain functional and metabolic properties of skeletal muscle may prevent or delay insulin resistance and type 2 diabetes.

Several distinct sets of transcription factors have been classified for a role in skeletal muscle atrophy, glucose metabolism, lipid metabolism, myogenesis, angiogenesis, and mitochondrial biogenesis [7], [8]. Notably, forkhead box proteins (FOXO), a family of transcription factors with FOXO1, FOXO3, FOXO4, and FOXO6 expressed in skeletal muscle, are implicated in a range of functions including regulation of muscle mass, fiber type specificity and metabolic flexibility [9], [10]. Understanding the role of FOXO isoforms in the regulation of insulin-stimulated gene expression and networks involved in substrate metabolism, mitochondrial function, and growth may provide insights into mechanisms controlling skeletal muscle plasticity in health and disease.

Overexpression of FOXO1 increases the percentage of fast twitch muscle fibers and decreases muscle size [11]. This phenotype is mirrored in skeletal muscle transiently expressing constitutively active FOXO3 [12], [13]. Inhibiting transcriptional activity of both FOXO1 and FOXO3 in skeletal muscle utilizing a FOXO construct lacking a transactivation domain (FOXOdn) increases fiber cross sectional area and myotube diameter in oxidative soleus muscle and glycolytic *tibialis anterior* muscle [14], [15]. Skeletal muscle-specific simultaneous deletion of FOXO1–3 isoforms, thereby avoiding any compensatory increase in other isoforms, attenuates anabolic signaling through Akt, and increases proteasomal degradation without affecting autophagic signaling [16]. Conversely, as the FOXO family is required for the induction of several atrophy-related genes, deletion of FOXO1–3 isoforms in skeletal muscle prevents the decline in muscle mass and force in response to fasting and denervation [17]. Collectively, these studies highlight a role for FOXO in skeletal muscle plasticity.

FOXO proteins play a role in the regulation of energy metabolism [10]. Perturbations that increase oxidative metabolism, including starvation and exercise, increase FOXO1 and FOXO3 protein abundance [18], [19], thereby associating the regulation of lipid metabolism with FOXO1/3 expression. Overexpression of FOXO1 in C2C12 myotubes increases protein abundance of fatty acid transporter protein CD36 [20] and lipoprotein lipase [19], and concomitantly decreases PDK4 and glycogen synthesis [20], further supporting a role for FOXO1 in oxidative metabolism. Conversely, *in vivo* ablation of FOXO1–4 does not alter muscle glycogen content [17]. Skeletal muscle-specific overexpression of FOXO1 in transgenic mice impairs glucose tolerance [11], without altering fed glucose levels [11], [21], implicating a role in glucose homeostasis. Nevertheless, the role of specific FOXO isoforms in metabolic homeostasis in skeletal muscle remains unclear. Of interest, glucocorticoids, anti-inflammatory hormones that regulate the switch from glycolytic to oxidative metabolism [22], upregulate FOXO1/3 expression in skeletal muscle [23], [24]. Thus, FOXO may play a transcriptional role in skeletal muscle to influence “*immunometabolism*” by altering processes governing immunological and metabolic processes [25].

The aim of this study was to elucidate the role of FOXO1 and FOXO3 transcriptional networks in skeletal muscle on glucose metabolism. This was achieved by transiently overexpressing FOXO constructs lacking the transactivation domain in mouse *tibialis anterior* muscle and determining the *in vivo* effects on glucose uptake, glycogen content, transcriptomic profiles, and relevant signaling pathways.

## 2 Materials and methods

### 2.1 Animal studies

Animal experiments were approved by the Regional Animal Ethical Committee (Stockholm, Sweden). Male C57BL/6J mice (30 week old) were purchased from Janvier (France). Mice received *ad libitum* access to water and standard rodent chow (Lantmännen, Sweden), and were housed on a 12 h light/dark cycle. Following one week of acclimatization, *tibialis anterior* muscle was transfected with either a control plasmid or plasmid encoding for FOXO1dn or FOXO3dn (Invitrogen GeneArt, ThermoFisher Scientific, Rockford, IL) by electroporation as described [26]. One week post-electroporation, mice were fasted for 4 h, and glucose uptake was measured *in vivo* using a modified oral glucose tolerance test as described [26]. Briefly, 4 h fasted mice received a glucose gavage (3 g/kg), and 2-[3H]deoxy-d-glucose (100 μl of saline/animal, 1 mCi/ml) was administered intraperitoneally. Mice were anesthetized with an intraperitoneal Avertin injection, 120 min after the start of the experiment, and electroporated *tibialis anterior* muscle was removed and rapidly frozen in liquid nitrogen. Glycogen content was determined using a commercially available kit (ab65620, Abcam, Cambridge, UK). A schematic representation of the animal experiments is shown in Figure S1.

### 2.2 Construct design

The FOXO1dn sequence was the same as previously described [27] consisting of amino acids 1–256. The FOXO3dn sequence was designed by aligning the murine amino acid sequence with a previously described dominant negative human sequence [28] yielding the 1–249 amino acid sequence. The FOXO1dn and FOXO3dn amino acid sequences obtained were optimized and converted to nucleotide sequences by GeneArt, and plasmids including LacZ encoding control vector were synthesized by GeneArt, (Invitrogen GeneArt, ThermoFisher Scientific). A schematic representation of the construct design can be found in Figure S1.

### 2.3 RNA extraction and gene expression analysis

qPCR analysis was performed on total RNA from skeletal muscle of mice that underwent an oral glucose tolerance test. RNA was extracted with Trizol (Life Technologies). Total RNA concentration was quantified spectrophotometrically (NanoDrop ND-1000 Spectrophotometer, ThermoFisher Scientific). RNA was reverse-transcribed to cDNA using the High Capacity cDNA RT kit (ThermoFisher Scientific) and gene expression was determined by real-time PCR utilizing SYBR Green reagents (Life Technologies, ThermoFisher Scientific). Gene expression was quantified with the ΔΔCt method using *Tbp* as control. Primer sequences are presented in Table 1. Microarray analysis was performed on total RNA extracted from electroporated muscle utilizing the EZ RNA extraction kit and hybridized to an Affymetrix Mouse Gene 2.1 ST array (ThermoFisher Scientific) at the core facility for Bioinformatics and Expression Analysis (BEA) at Karolinska Institutet. The microarray data are publicly available at Gene Expression Omnibus (GEO accession: GSE105778).

**Table 1:** Primer sequences.

Gene	Forward primer	Reverse primer
*Foxo1*	CTGCAGATCCCGTAAGACG	GGTCACCGGTGTCTAAGGAG
*Foxo3*	GGAAGGGAGGAGGAGGAATG	CTCGGCTCCTTCCCTTCAG
*Ccl2*	AGCCAACTCTCACTGAAGCC	TTCTTGGGGTCAGCACAGAC
*Ccl7*	CCACCATGAGGATCTCTGCC	ATAGCCTCCTCGACCCACTT
*Ccl8*	TTTGCCTGCTGCTCATAGCT	TGTGAAGGTTCAAGGCTGCA
*Cxcl9*	ACCTCAAACAGTTTGCCCCA	ACGACGACTTTGGGGTGTTT
*Cd68*	AAGGTCCAGGGAGGTTGTGA	ATGAATGTCCACTGTGCTGC
*Cd48*	CTCGGGACCTTTCCCCAAAA	ACTAGCCAAGTTGCAGTCCA
*Itgax*	CCAGCCAGAGGATTTCAGCAT	CTGCAGGTGTGAAGTGAACAG
*Cd3g*	ACTGTAGCCCAGACAAATAAAGC	TGCCCAGATTCCATGTGTTTT
*Ncr1*	GAGCCAGAGGATCAACACTG	ATGGCTTTGGTCTCTCCAAGG
*Ly6c*	ACCCTTCTCTGAGGATGGACA	GCTGGGCAGGAAGTCTCAAT
*Tbp*	CCTTGTACCCTTCACCAATGAC	ACAGCCAAGATTCACGGTAGA

### 2.4 Immunoblot analysis

Western blot analysis was performed as described from skeletal muscle of mice that underwent an oral glucose tolerance test [26]. Ponceau staining was used to confirm equal protein loading [29]. The following antibodies used for immunoblot analysis were purchased from Cell Signaling Technology (Beverly, MA): Akt (#9272), p-Akt Thr^308^ (#4056), p-Akt Ser^473^ (#9271), GSK3β (#9315), p-GSK3β Ser^9^ (#9323), GS (#3839), p-GS Ser^641^ (#3891), mTOR (#2983), p-mTOR Ser^2448^ (#5536), 4EBP1 (#9644), p-4EBP1 Thr^37/46^ (#2855), p-p70S6K Thr^389^ and Thr^421^/Ser^424^ (#2708), p70S6K (#9205), p-STAT1 Tyr^701^ (#9171), STAT1 (#9172). The following antibodies were purchased from Abcam (Cambridge, UK): total OXPHOS Rodent WB Antibody Cocktail (ab110413), FOXO1 (ab12161), and FOXO3 (ab47409). Antibodies against GLUT4 (#07-1404, Millipore, Darmstadt, Germany) and Hexokinase 2 (kindly provided by Oluf Pedersen, University of Copenhagen) were used. Appropriate secondary mouse or rabbit antibodies were purchased from Bio-Rad. The immunoreactive proteins were quantified densitometrically utilizing Quantity One Software (Bio-Rad).

### 2.5 Statistical analysis

CEL files from the microarray experiment on platform Affymetrix MoGene-2_1-st were collected for 41345 probesets and 24 samples (6 paired samples with control and FOXO1dn overexpression construct, 6 paired samples with control and FOXO3dn overexpression construct). Data was analyzed in R (version 3.4.4) [30] utilizing the oligo package for the robust multi-array average (RMA) normalization using the oligo library from Bioconductor [31] on the pd.mogene.2.1.st library [32]. Transcripts with miRNA, pseudogene, and predicted genes, were filtered out from the dataset since a single probe was overlapping with one or more of these annotated regions, reducing the probeset to 22,557. Probesets belonging to multiple genes were collapsed by using collapseRows function from WGCNA R package [33]. This procedure resulted in 20,295 unique genes for downstream analysis.

Differential expression analysis was performed with limma [34], and sample pairing was defined with duplicateCorrelation, using the model design ‘y ∼ 0 + plasmid’, where plasmid represented whether mice were electroporated with control or FOXO construct. Transcriptome data was visualized using the factoextra and ggplot2 package [35]. Gene set enrichment analysis (GSEA) was performed with clusterProfiler [36], with the minimum gene set size 10, and 1,000,000 permutations for GSEA. Inflammatory cell signature deconvolution analysis was performed using the CIBERSOFT framework [37], using murine immune cell signatures [38].

Glucose uptake, qPCR and western blot data were analyzed in GraphPad Prism 7 (GraphPad Software, Inc., La Jolla, CA, USA) with a paired student *t*-test. The significance threshold was defined at *p* < 0.05, except for the transcriptomic analysis, where significance was defined at a multiple testing adjusted *p*-value < 0.05 and absolute log_2_ fold-change > 1.

## 3 Results

### 3.1 Glucose uptake and glycogen content

**Figure 1:**
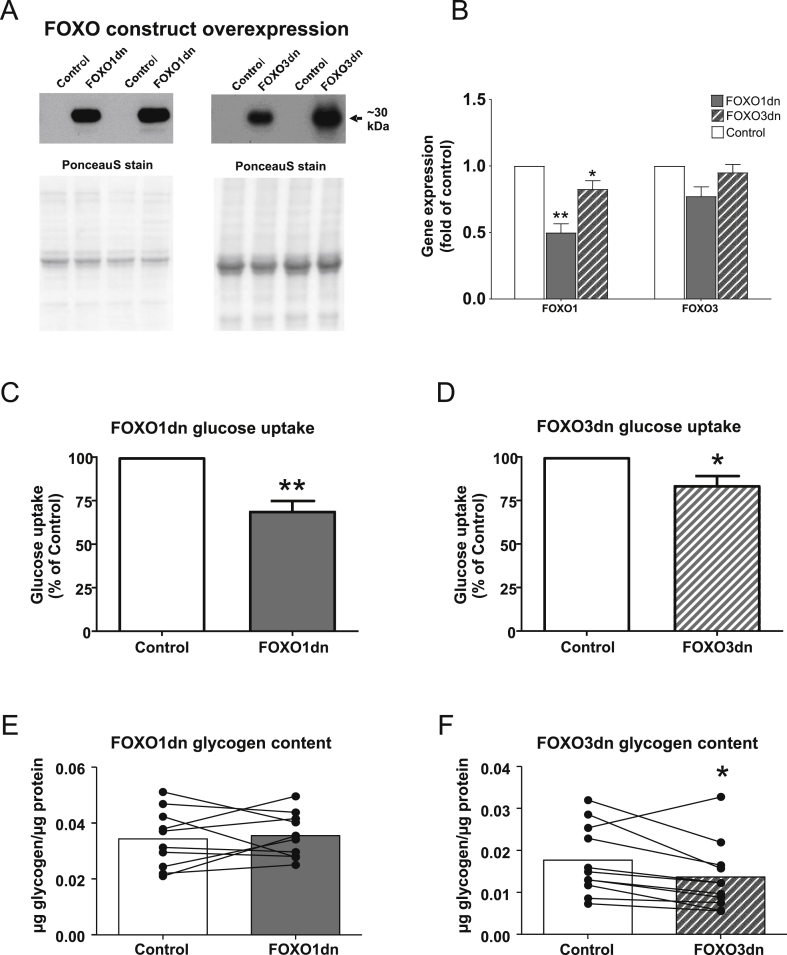
***In vivo*****glucose uptake and glycogen content in*****tibialis anterior*****skeletal muscle overexpressing FOXO1dn or FOXO3dn constructs**. (A) Representative western blot of FOXO1dn and FOXO3dn construct overexpression. (B) Endogenous FOXO1 and FOXO3 expression 7 days following electroporation with either FOXO1dn or FOXO3dn constructs detected by qPCR. (C) *In vivo* glucose uptake during a 2-h oral glucose tolerance test (3 g/kg) measured by accumulation of ^3^H-deoxyglucose in skeletal muscle after FOXO1dn versus respective contralateral control leg, or (D) FOXO3dn versus respective contralateral leg. (E) Glycogen content in skeletal muscle after FOXO1dn versus respective contralateral control leg, or (F) FOXO3dn versus contralateral leg. Data are mean or individual fold changes ± SEM for paired muscle samples. *n* = 12 mice per construct, *p < 0.05 and **p < 0.01.

Electroporation of *tibialis anterior* muscle with either FOXO1dn or FOXO3dn constructs (Figure S1) led to efficient overexpression of each respective protein as detected by western blot analysis (Figure 1A). FOXO1dn and FOXO3dn electroporation led to changes in gene expression of canonical FOXO responsive genes [17] (Figure S2A,B). FOXO1dn transfection decreased endogenous FOXO1 expression 50% (*p* < 0.01) and endogenous FOXO3 expression 20% (*p* < 0.05), while FOXO3dn transfection was without effect on either endogenous isoforms (Figure 1B). Overexpression of either FOXO1dn (Figure 1C) or FOXO3dn (Figure 1D) construct decreased *in vivo* glucose uptake during a glucose tolerance test, as compared to the contralateral control muscle (35%, *p* < 0.001 and 20%, *p* < 0.05, for FOXO1dn and FOXO3dn, respectively). Intramuscular glycogen content was unaltered by overexpression of the FOXO1dn construct (Figure 1E) and decreased 20% by overexpression of the FOXO3dn construct (*p* < 0.05, Figure 1F).

### 3.2 Transcriptomic analysis

**Figure 2:**
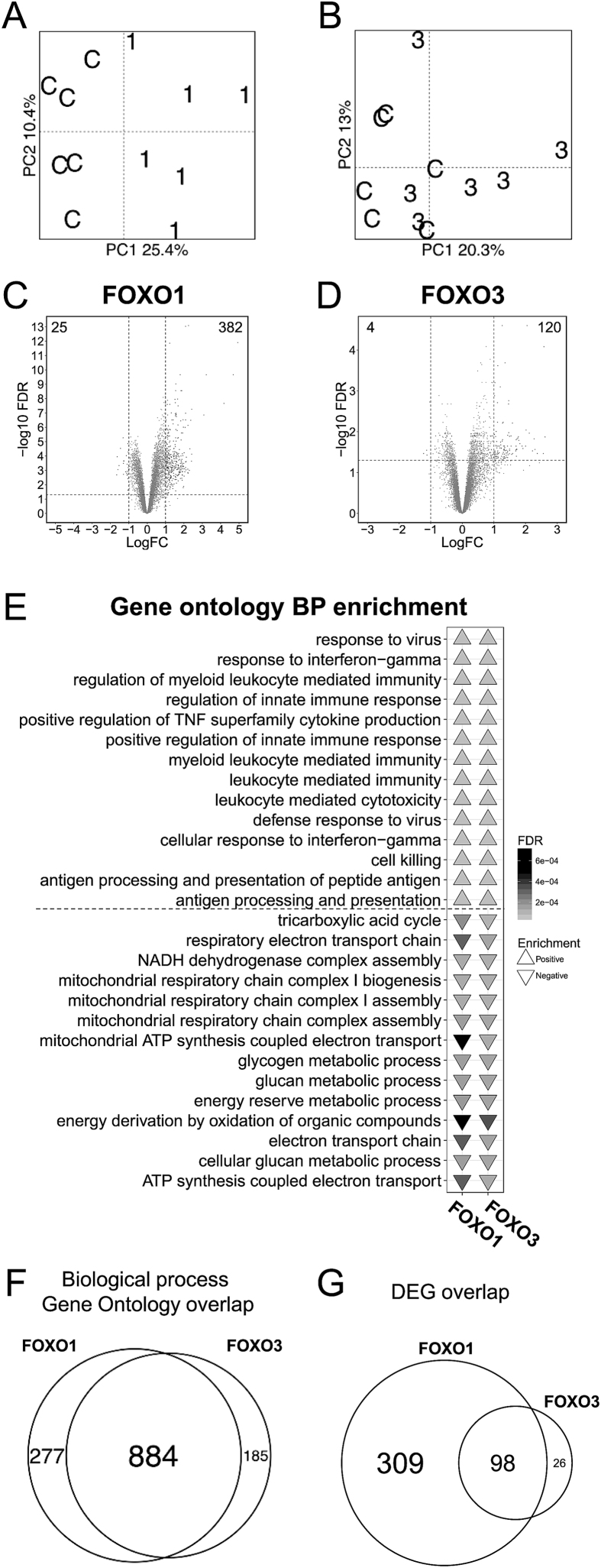
**Transcriptomic analysis of *tibialis anterior* muscle after FOXOdn overexpression**. (A) Principal component analysis of transcriptomic data of *tibialis anterior* muscle after FOXO1dn or (B) FOXO3dn overexpression. (C) Volcano plot showing changes in gene expression after FOXO1dn or (D) FOXO3dn overexpression. Dashed lines indicate value cutoff at multiple testing adjusted *p* < 0.05 and fold change of at least ±1 logFC. (E) Overlap of FOXO1 and FOXO3 GSEA, showing biological process (BP) gene ontologies, with the triangle showing negative or positive enrichment, and the shade indicating FDR. (F) Overlap of significantly enriched BP gene ontologies after FOXO1dn or FOXO3dn overexpression. (G) Overlap of differentially expressed genes in *tibialis anterior* muscle after FOXO1dn or FOXO3dn overexpression. *n* = 6 mice, all indicated pathways are significant at FDR < 0.05.

Transcriptomic and principal component analysis revealed that the insulin-stimulated gene expression profiles are clearly separated between FOXO1dn transfected and control plasmid transfected muscle (Figure 2A). Conversely, the separation of gene expression profiles between FOXO3dn transfected and control plasmid transfected muscle was less obvious (Figure 2B). FOXO1dn transfection downregulated the expression of 25 genes and upregulated the expression of 382 genes (Figure 2C), while FOXO3dn transfection downregulated the expression of 4 genes and upregulated the expression of 120 genes (Figure 2D). Expression results are reported in Supplementary Table 1. Gene set enrichment analysis of the FOXO1dn- or FOXO3dn-transfected skeletal muscle transcriptome revealed that the top positively enriched gene ontologies were associated with inflammatory processes, while the top negatively enriched gene ontologies were associated with energy metabolism (Figure 2E, Supplementary Table 2). The overall overlap of enriched gene ontologies by each construct was substantial, with 884 common, and 277 unique for FOXO1dn and 185 unique for FOXO3dn transfection (Figure 2F). KEGG pathway GSEA of FOXO1dn- or FOXO3dn-transfected skeletal muscle revealed a similar response on the transcriptome between the two constructs (Figure S3A, Supplementary Table 3), and the overall overlap of KEGG pathways was also substantial (Figure S3B). The overlap was 309 unique genes for FOXO1, 26 for FOXO3, and 98 in common (Figure 2G). The overlap of differentially expressed genes for each construct and publicly available data of chromatin immunoprecipitation data from FOXO1 [39] and FOXO3 [40] was 2 and 6 respectively (Figure S4A,B).

### 3.3 Protein abundance of GLUT4 and mitochondrial oxidative phosphorylation (OXPHOS) complexes

**Figure 3:**
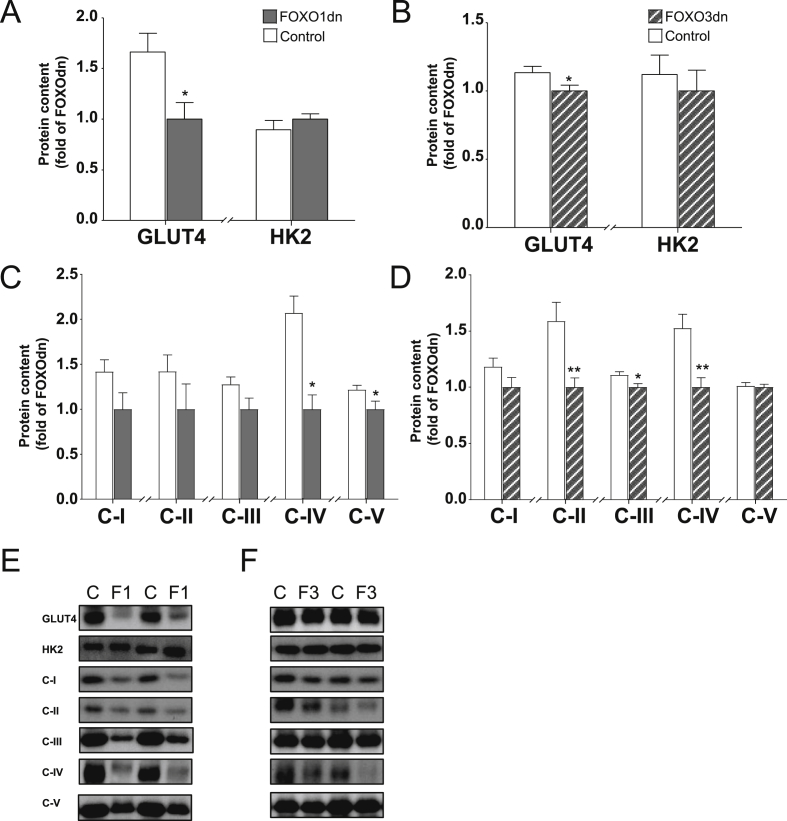
**FOXOdn transfection effects on abundance of proteins involved in glucose metabolism and oxidative phosphorylation in skeletal muscle**. (A) Quantification of GLUT4 and Hexokinase 2 (HK2) protein content in skeletal muscle after FOXO1dn transfection, or (B) FOXO3dn transfection. (C) Quantification of protein abundance of mitochondrial oxidative phosphorylation chain enzymes, complex I–V, following FOXO1dn transfection, or (D) FOXO3dn transfection. (E) Representative western blots of GLUT4, HK2 and the mitochondrial respiratory chain complex protein abundance. Data are mean ± SEM. *n* = 11 mice, *p < 0.05 and **p < 0.01.

GLUT4 protein abundance was decreased in response to overexpression of either the FOXO1dn (40%, *p* < 0.05; Figure 3A,E) or the FOXO3dn (10%, *p* < 0.05; Figure 3B,E) construct, while HK2 protein content was unaffected (Figure 3A,B,E). Overexpression of either the FOXO1dn or the FOXO3dn construct attenuated the abundance of several proteins involved in oxidative phosphorylation. FOXO1dn overexpression decreased protein abundance of complex IV and complex V (50%, *p* < 0.05 and 20%, *p* < 0.05 respectively; Figure 3C,E), and FOXO3dn overexpression decreased protein abundance of complex II, III, and IV (40%, *p* < 0.001; 10%, *p* < 0.05; and 30%, *p* < 0.01 respectively; Figure 3D,E). Gene expression of complex IV subunits was decreased in 8 and increased in 2 out of 24 subunits measured after FOXO1dn overexpression, while FOXO3dn overexpression decreased the expression of only 1 subunit (Figure S4A,B).

### 3.4 Akt and mTOR signaling

**Figure 4:**
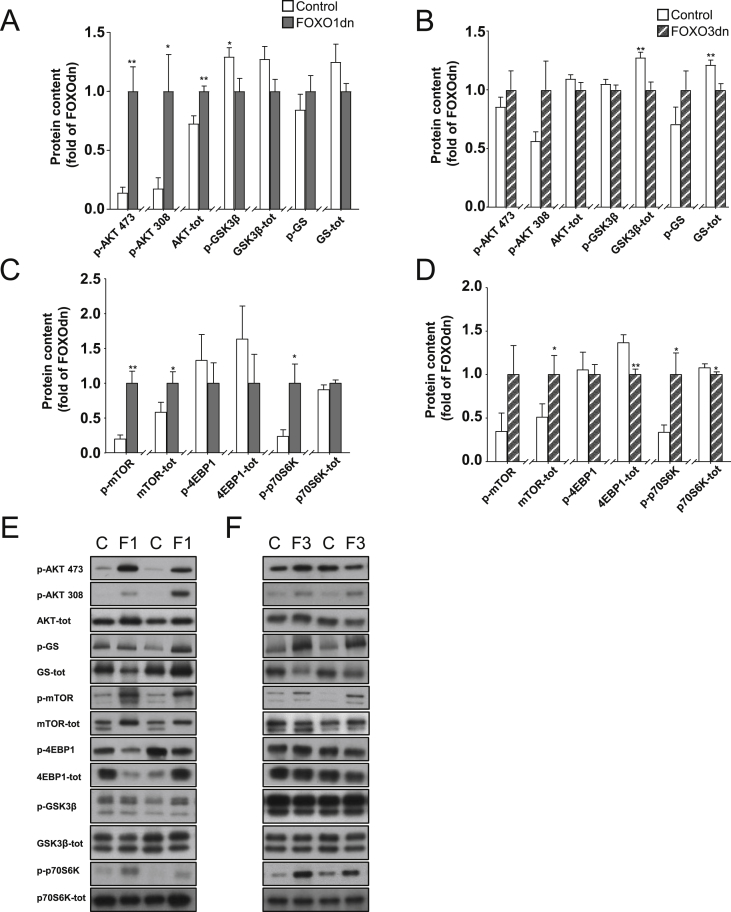
**Changes in total protein abundance and phosphorylation of signaling pathways in *tibialis anterior* muscle after FOXO1dn or FOXO3dn overexpression**. (A) Quantification of protein abundance and phosphorylation of Akt signaling in *tibialis anterior* skeletal muscle after FOXO1dn transfection, or (B) FOXO3dn transfection. (C) Quantification of protein abundance and phosphorylation of mTOR, and protein synthesis signaling following FOXO1dn transfection, or (D) FOXO3dn transfection. (E) Representative western blots of the studied signaling cascades for FOXO1dn transfection, or (F) FOXO3dn transfection. Data are mean ± SEM. *n* = 11 mice, *p < 0.05 and **p < 0.01.

Overexpression of the FOXO1dn construct increased Akt signaling in skeletal muscle, as evidenced by increased Akt protein (140%, *p* < 0.001), p-Akt Ser^473^ (720%, *p* < 0.05) and p-Akt Thr^308^ (570%, *p* < 0.005; Figure 4A,E), whereas overexpression of the FOXO3dn construct was without effect (Figure 4B,F). Overexpression of the FOXO3dn construct decreased glycogen synthase protein abundance (20%, *p* < 0.01), without altering glycogen synthase phosphorylation (Figure 4B,F), whereas overexpression of the FOXO1dn construct had no effect (Figure 4A). GSK3β phosphorylation was decreased by FOXO1dn transfection (20%, *p* < 0.05), while FOXO3dn transfection decreased GSK3β total protein content (20%, *p* < 0.01) (Figure 4A,B,E,F).

Skeletal muscle overexpression of either the FOXO1dn or FOXO3dn construct increased mTOR protein abundance (170% and 190%, *p* < 0.05), while phosphorylation was only increased by FOXO1dn transfection (500%, *p* < 0.0005) (Figure 4C–F). The mTOR target, p70S6K, was modestly decreased (7%, *p* < 0.05) in response to FOXO1dn transfection (Figure 4C,E), and unaltered in response to FOXO3dn transfection (Figure 4D,F), while p70S6K phosphorylation was increased (420%, *p* < 0.05 and 300%, *p* < 0.05 respectively; Figure 4C–F). Total 4E-BP1 protein abundance decreased (30%, *p* < 0.005) only in response to the FOXO3dn transfection (Figure 4D,F).

### 3.5 Inflammatory signaling

**Figure 5:**
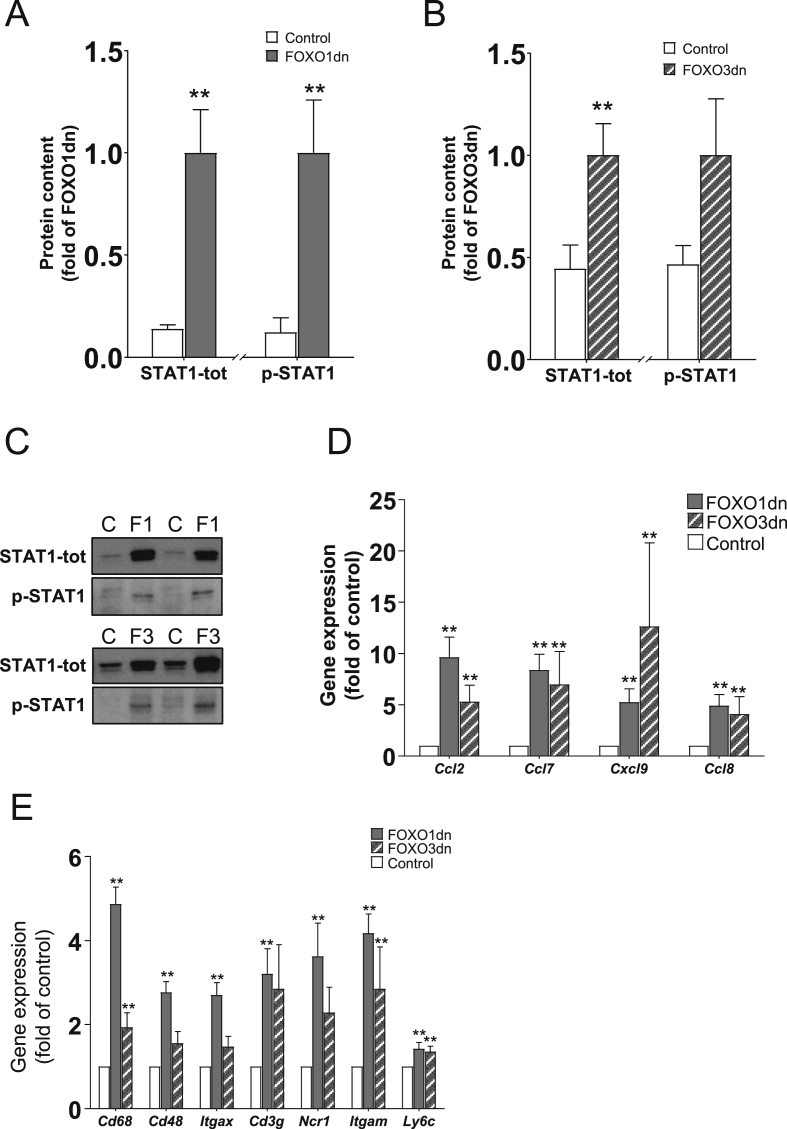
**Effects of FOXOdn overexpression on inflammatory signaling pathways in *tibialis anterior* muscle**. (A) Quantification of STAT1 protein content and phosphorylation after FOXO1dn or (B) FOXO3dn electroporation in *tibialis anterior* muscle. (C) Representative western blots of STAT1 protein content and phosphorylation after FOXO1dn or FOXO3dn overexpression. (D) Gene expression analysis of chemokine genes after FOXO1dn or FOXO3dn overexpression. (E) Gene expression of key immune cell markers after FOXO1dn or FOXO3dn overexpression. Data are mean ± SEM. *n* = 11 mice. *p < 0.05 and **p < 0.01.

Protein content of STAT1 was increased in skeletal muscle in response to overexpression of either the FOXO1dn or FOXO3dn construct (720%, *p* < 0.005 and 220%, *p* < 0.05 respectively; Figure 5A–C), whereas STAT1 phosphorylation was increased only in response to the FOXO1dn transfection (820%, *p* < 0.01) (Figure 5A,C). Gene expression of the chemokines *Ccl2*, *Ccl7*, *Cxcl9*, and *Ccl8*, were robustly upregulated in response to overexpression of either construct (Figure 5D). Markers of immune cells, including *Cd68*, *Cd48*, *Itgax*, *Cd3g*, *Ncr1*, *Itgam*, and *Ly6c*, were increased by FOXO1dn transfection (*p* < 0.01). FOXO3dn transfection increased *Cd68* (200%, *p* < 0.01), *Itgam* (170%, *p* < 0.05) and *Ly6c* (130%, *p* < 0.05) mRNA expression (Figure 5E). Immune cell signature analysis showed that the M1 macrophages were the main signature enriched in response to either FOXO1dn or FOXO3dn transfection (Figure S6A,B). Moreover, the immune cell signatures were efficiently separated between FOXO1dn and FOXO3dn transfected samples from the respective control samples using principal component analysis (Figure S6C,D).

## 4 Discussion

FOXO transcription factors regulate skeletal muscle mass and fiber type characteristics [9], [10]. Furthermore, FOXO proteins are essential for a wide range of metabolic functions, including the control of carbohydrate and lipid oxidation in skeletal muscle during fasting [18] or high fat feeding [41]. Here we provide evidence that overexpression of either a FOXO1dn or FOXO3dn construct in skeletal muscle attenuates glucose uptake. FOXO transcriptional regulation of glucose uptake is likely to involve several mechanisms. We found FOXO1dn or FOXO3dn transfection decreased GLUT4 protein abundance, which may account for the attenuation of glucose uptake [42]. The reduction in GLUT4 protein may occur from the regulation of PPARγ1 or PPARγ2 promoter binding [43], or by a direct interaction between FOXO and the GLUT4 promoter [44]. Skeletal muscle specific triple deletion of FOXO1, FOXO,3 and FOXO4, does not affect glycogen content [17], implying that the binding sites of FOXO proteins might be relevant for other transcription factors that regulate metabolism. However, we found overexpression of FOXO3dn, but not FOXO1dn, decreased intramuscular glycogen content. Thus, our results indicate that glucose storage is under the regulation of FOXO3 transcriptional activity and implies specialized roles of FOXO isoforms in the control of glucose uptake and metabolism.

FOXO proteins regulate energy homeostasis through the control of glucose metabolism and mitochondrial respiration [10]. Liver specific deletion of FOXO1 restores mitochondrial respiration in insulin resistant mice [45], while *in vitro* expression of constitutively active FOXO3 reduces mitochondrial respiration and respiratory chain complex proteins [46]. FOXO1 activation in C2C12 skeletal muscle cells increases fatty acid uptake and oxidation and drives the expression of genes involved in lipid metabolism [20]. This finding suggests that inactivation of FOXO1 may contribute to the accumulation of intramuscular lipids and insulin resistance. Here we found that a transient inhibition of FOXO transcriptional activity decreased the abundance of mitochondrial proteins in skeletal muscle. Thus, the reduction in glucose uptake in skeletal muscle overexpressing FOXO1dn or FOXO3dn may arise from an accumulation of intramuscular lipids or metabolic intermediates due to attenuated mitochondrial respiration. These changes may occur by a direct or indirect gene regulatory mechanism, or secondarily from reduced glucose uptake. Furthermore, the decreased GLUT4 protein content, combined with the decreased abundance of mitochondrial OXPHOS complexes, may account for the reduction in glucose uptake in skeletal muscle. Skeletal muscle glycogen content and glycogen synthase protein content, was decreased only after FOXO3dn transfection, indicating that FOXO3 plays a role in regulating glucose storage. The decrease in OXPHOS complexes and GLUT4 after FOXO1dn transfection are insufficient to affect skeletal muscle glycogen content as observed after FOXO3dn transfection.

We performed a transcriptomic analysis to identify distinct signatures of gene expression profiles influenced by inactivation of FOXO1 or FOXO3 in skeletal muscle. The microarray analysis was validated by qPCR (Figure 5D,E). Canonical FOXO target genes [17] were changed by FOXOdn transfection (Figure S2A,B), indicating that the inhibition of FOXO transcriptional activity was successful. FOXO1dn transfection had more robust effects on FOXO target genes as compared with FOXO3dn, reflecting the results from the differential expression analysis. Our transcriptome analysis revealed that the overlap of differentially expressed genes induced by the FOXO1dn and FOXO3dn overexpression was ∼10% and 90% respectively. The pathways modulated by the FOXO1dn and FOXO3dn transfections were remarkably similar, with ∼80% being shared. The transcriptomic changes observed after FOXOdn transfections appear to be secondary, as there was little overlap with publicly available chromatin immunoprecipitation data [39], [40]. This would suggest that the function of FOXO proteins on metabolism and inflammation is to coordinate the gene expression response to environmental stimuli.

Overexpression of FOXO1dn or FOXO3dn constructs reduces the expression of genes controlling oxidative phosphorylation and fatty acid metabolism. These results were consistent with changes in the abundance of mitochondrial complex proteins, and decreases in the expression of genes encoding mitochondrial complex proteins, implying that the changes are due to transcriptional, rather than posttranslational effects. Moreover, we found the Akt signaling pathway as well as several inflammatory pathways were altered in skeletal muscle overexpressing FOXO1dn or FOXO3dn constructs. Thus, inactivation of FOXO signaling gives rise to an *immunometabolism* gene signature that is characteristic of obesity-induced insulin resistant states [47], [48]. Inflammatory signaling alters lipid metabolism in liver, adipose tissue, skeletal muscle, and macrophages in the context of infection, diabetes, and atherosclerosis.

FOXOdn transfection attenuated skeletal muscle glucose uptake, concomitant with increased Akt signaling, as revealed by our protein content and phosphorylation analysis. Additionally, FOXOdn transfection increased Akt signaling, consistent with our finding of increased mTOR protein content and p70S6K phosphorylation after transfection with either FOXO1dn or FOXO3dn. Previous studies establish that FOXO proteins regulate insulin sensitivity in liver [49], [50], skeletal muscle [51], and adipose tissue [52]. Furthermore, FOXO1 has been shown to increase Akt phosphorylation through tribble 3, a modulator of Akt activity, by suppressing its promotor activity [50], and stimulate phosphatase activity in cardiomyocytes [53]. Here we show that inhibition of FOXO1 transcriptional activity increased Akt phosphorylation, indicating that FOXO transcription binding sites rather than FOXO proteins are likely to mediate tribble 3 or phosphatase activity. Thus, the two contrasting observations of decreased glucose uptake (due to decreased protein content of GLUT4 and energy metabolizing enzymes), and increased Akt phosphorylation (due to changes in Akt modulating enzymes) might be due independent and parallel mechanisms.

FOXO proteins regulate inflammatory cell function [54]. FOXO1 or FOXO3 deficiency *in vivo* leads to immune cell activation and proliferation [55], [56]. Our GSEA indicates that several inflammatory signaling pathways are enriched after either FOXO1dn or FOXO3dn transfection. This observation was biochemically validated by measuring changes in chemokine expression, STAT1 signaling, and immune cell markers. Moreover, our data suggest that FOXO1 and FOXO3 transcriptional activity is necessary for suppression of inflammatory signaling, as STAT1 total protein content, expression of chemoattractants, and markers of immune cells were robustly increased in skeletal muscle overexpressing FOXO1dn or FOXO3dn constructs. The increased STAT1 protein content after FOXO1dn or FOXO3dn transfection suggests that interferon γ and interferon β signaling was increased after transfection [57], in accordance with the predictions from the pathway analysis. The increased expression of chemokines is concurrent with increased expression of immune cell markers, confirming that FOXO transcriptional activity is involved in the recruitment of inflammatory cells within skeletal muscle. However, as the electroporation procedure could cause early and transient inflammation [58], FOXO transcriptional activity might be necessary for suppressing an initial inflammatory insult. Muscle inflammation regulates glucose uptake and metabolism [59], [60], raising the possibility that tissue inflammation occurs synergistically with changes in mitochondrial and GLUT4 protein levels to regulate glucose handling after FOXOdn transfection. The possibility that FOXOdn transfection might increase systemic inflammation through secreted chemokines seems implausible given that the control transfected leg showed lower levels of inflammatory signaling. Future studies are warranted to establish the mechanism by which FOXO transcriptional activity governs inflammation in skeletal muscle, and whether FOXO regulation of inflammatory processes is independent of glucose and energy homeostasis.

FOXO transcriptional activity is increased in several conditions, including skeletal muscle insulin resistance, exercise, and atrophy, highlighting the role of FOXO as a central transcriptional regulator of maintenance of skeletal muscle energy homeostasis [9], [10], [12]. Our study reveals that FOXO1 and FOXO3 transcriptional activity is necessary for the regulation of glucose handling and control of inflammatory signaling in mature skeletal muscle. A further understanding of the role of FOXO in the control of metabolic and inflammatory events in skeletal muscle may identify pathways governing “immunometabolic” networks involved in several pathophysiological conditions affecting skeletal muscle function.
