# Supplementary material for: Regulation of glucose uptake and inflammation markers by FOXO1 and FOXO3 in skeletal muscle
Source: Mol Metab. 2018 Nov 16;20:79–88. doi: 10.1016/j.molmet.2018.09.011 (PMC6358548; doi:10.1016/j.molmet.2018.09.011)
Supplement: Multimedia component 4 [file mmc4.pptx]

## Slide 1
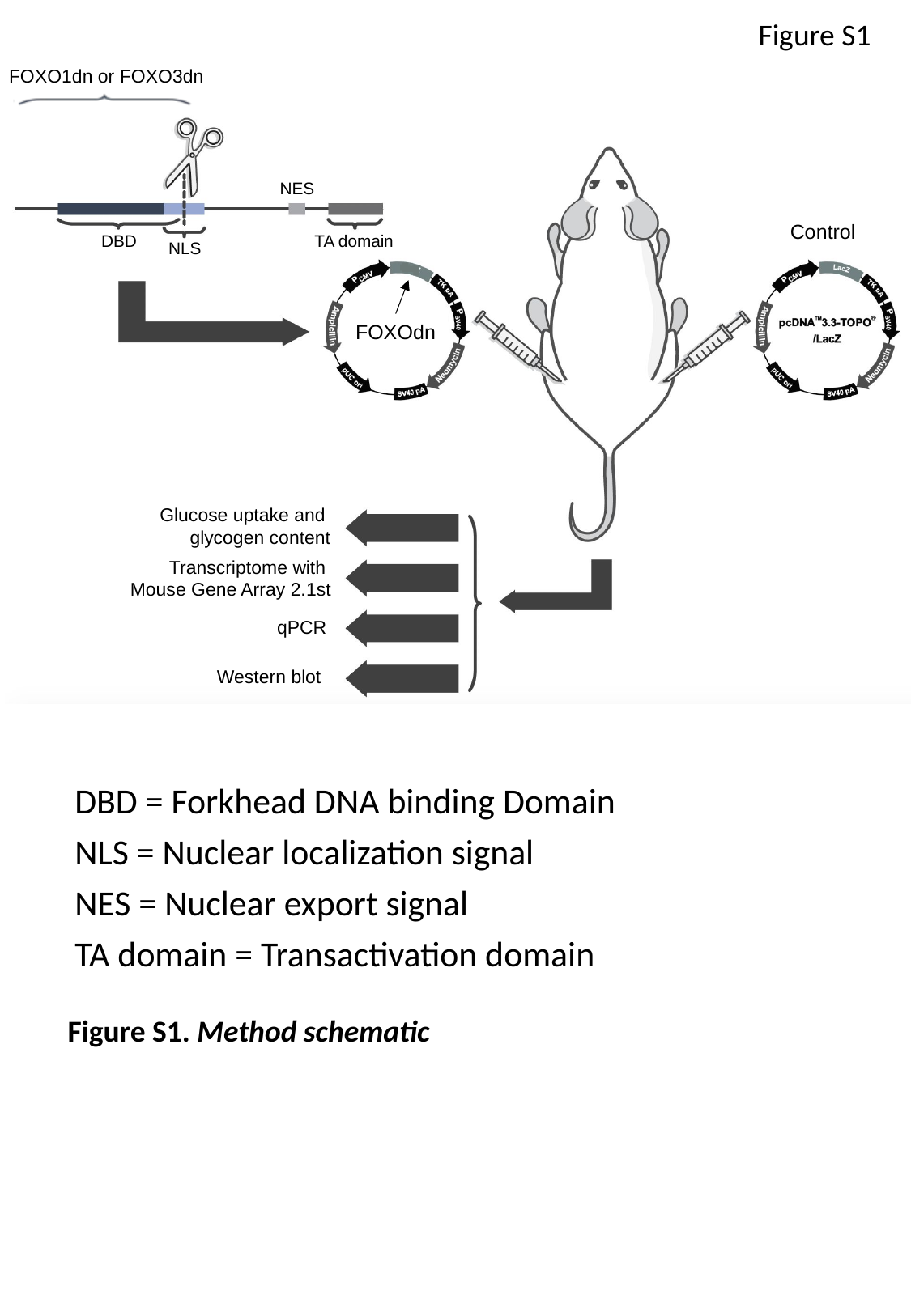

Figure S1
FOXO1dn or FOXO3dn
NES
Control
DBD
TA domain
NLS
FOXOdn
Glucose uptake and
glycogen content
Transcriptome with
Mouse Gene Array 2.1st
qPCR
Western blot
DBD = Forkhead DNA binding Domain
NLS = Nuclear localization signal
NES = Nuclear export signal
TA domain = Transactivation domain
Figure S1. Method schematic

## Slide 2
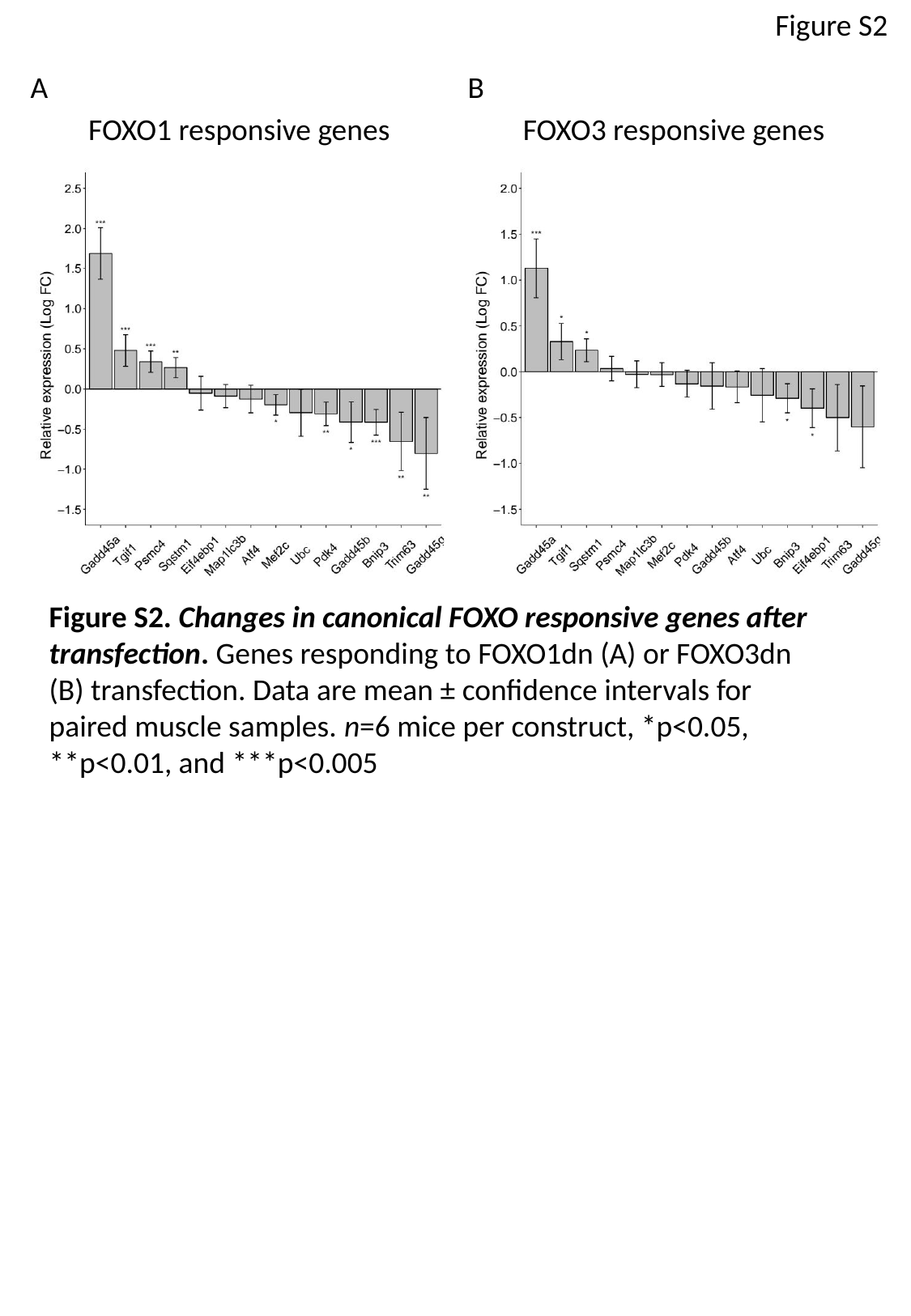

Figure S2
A
B
FOXO1 responsive genes
FOXO3 responsive genes
Figure S2. Changes in canonical FOXO responsive genes after transfection. Genes responding to FOXO1dn (A) or FOXO3dn (B) transfection. Data are mean ± confidence intervals for paired muscle samples. n=6 mice per construct, *p<0.05, **p<0.01, and ***p<0.005

## Slide 3
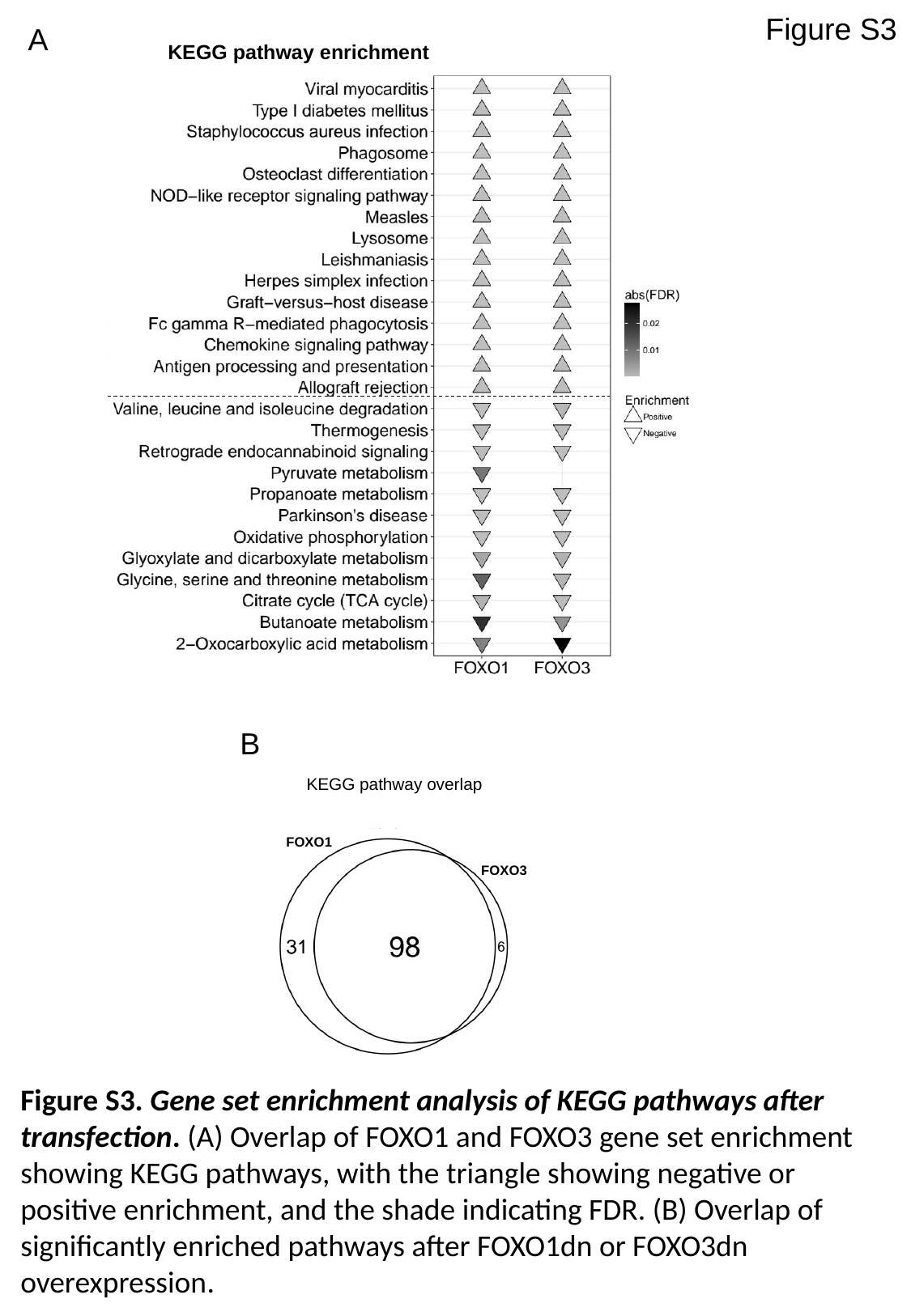

Figure S3
A
KEGG pathway enrichment
B
KEGG pathway overlap
FOXO1
FOXO3
Figure S3. Gene set enrichment analysis of KEGG pathways after transfection. (A) Overlap of FOXO1 and FOXO3 gene set enrichment showing KEGG pathways, with the triangle showing negative or positive enrichment, and the shade indicating FDR. (B) Overlap of significantly enriched pathways after FOXO1dn or FOXO3dn overexpression.

## Slide 4
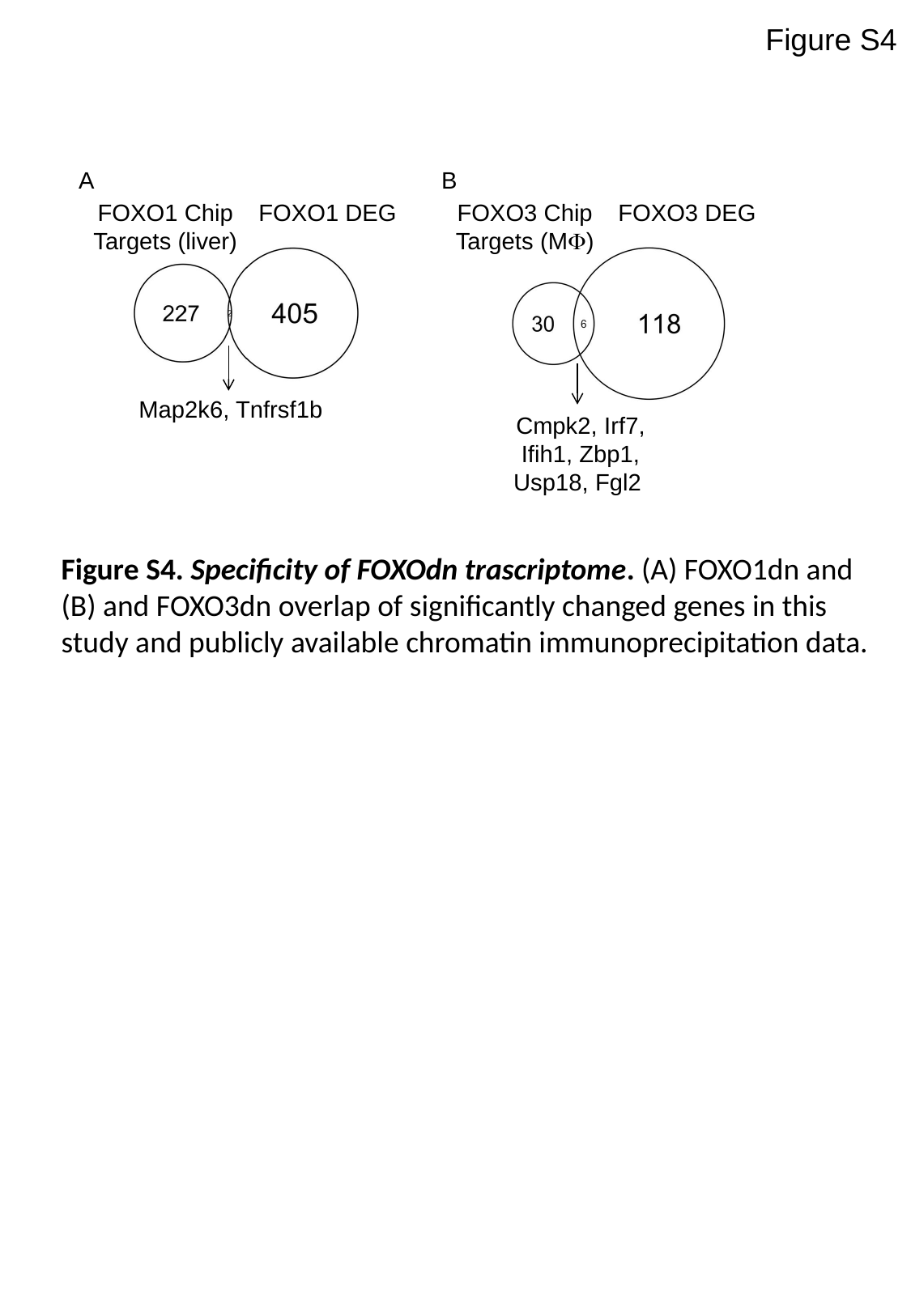

Figure S4
A
B
FOXO1 Chip
Targets (liver)
FOXO1 DEG
FOXO3 Chip
Targets (M)
FOXO3 DEG
Map2k6, Tnfrsf1b
Cmpk2, Irf7, Ifih1, Zbp1, Usp18, Fgl2
Figure S4. Specificity of FOXOdn trascriptome. (A) FOXO1dn and (B) and FOXO3dn overlap of significantly changed genes in this study and publicly available chromatin immunoprecipitation data.

## Slide 5
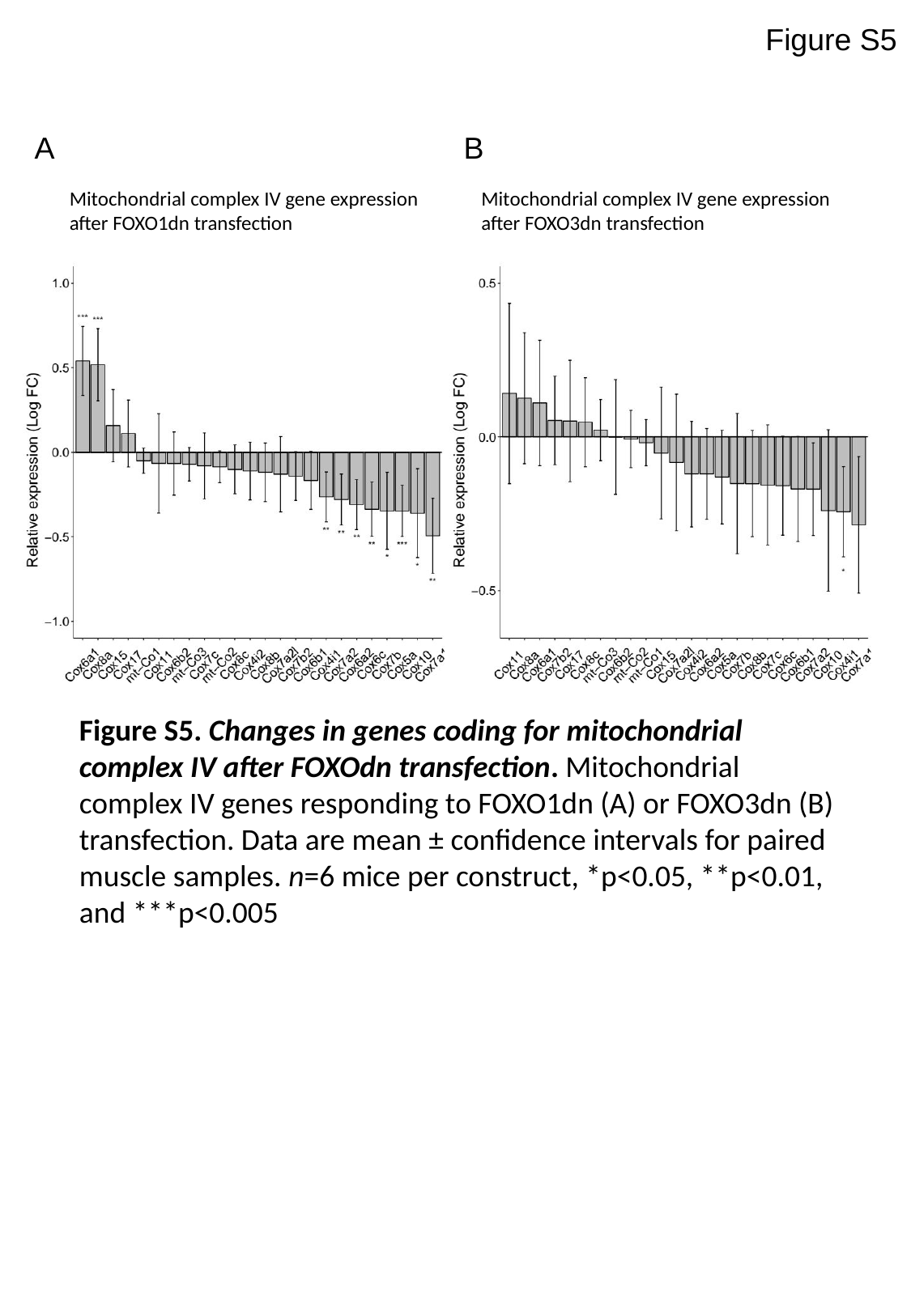

Figure S5
A
B
Mitochondrial complex IV gene expression
after FOXO1dn transfection
Mitochondrial complex IV gene expression
after FOXO3dn transfection
Figure S5. Changes in genes coding for mitochondrial complex IV after FOXOdn transfection. Mitochondrial complex IV genes responding to FOXO1dn (A) or FOXO3dn (B) transfection. Data are mean ± confidence intervals for paired muscle samples. n=6 mice per construct, *p<0.05, **p<0.01, and ***p<0.005

## Slide 6
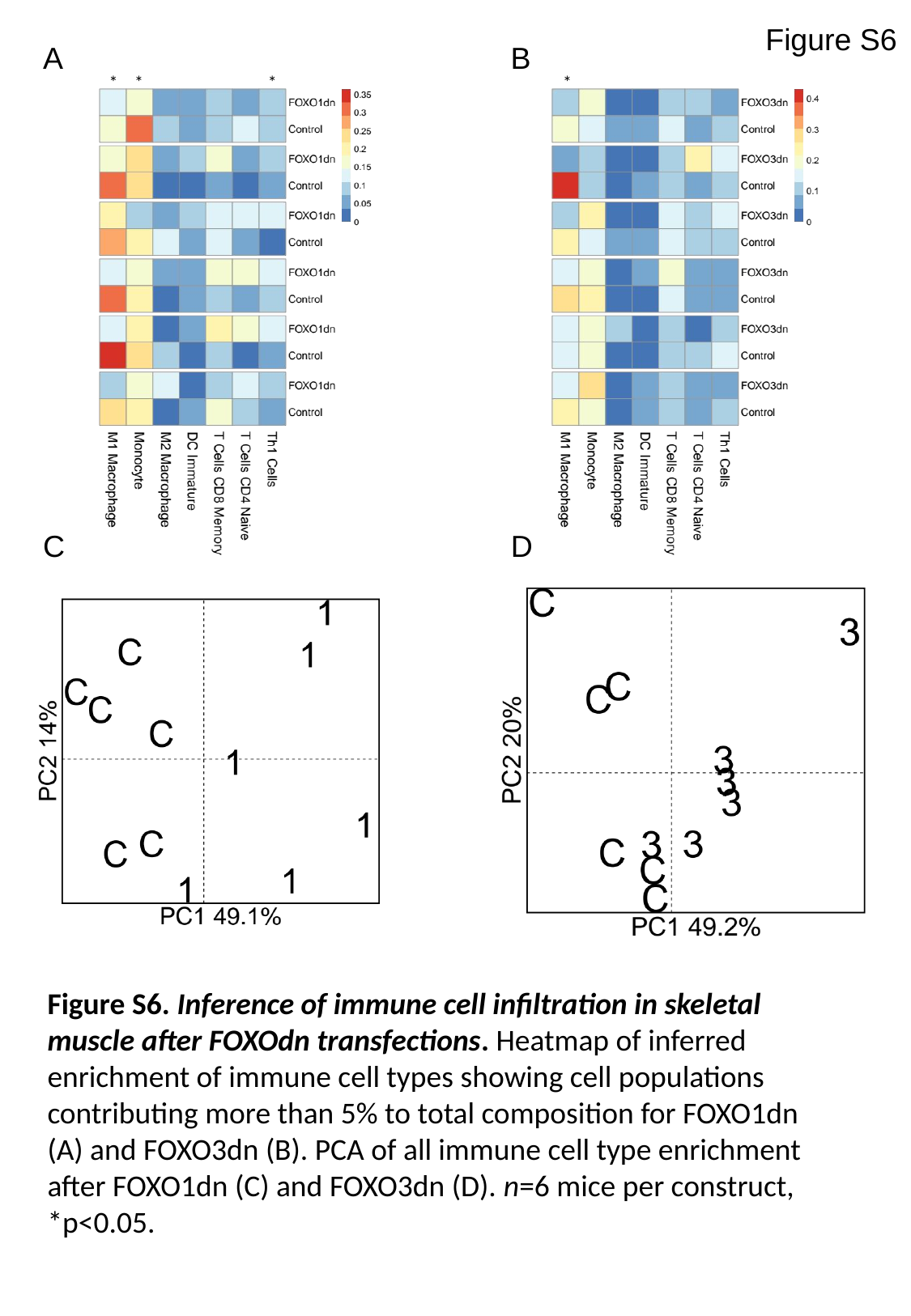

Figure S6
A
B
*
*
*
*
C
D
Figure S6. Inference of immune cell infiltration in skeletal muscle after FOXOdn transfections. Heatmap of inferred enrichment of immune cell types showing cell populations contributing more than 5% to total composition for FOXO1dn (A) and FOXO3dn (B). PCA of all immune cell type enrichment after FOXO1dn (C) and FOXO3dn (D). n=6 mice per construct, *p<0.05.
